# Surgical Techniques in Nontraumatic Midcarpal Instability: Evaluating the Dorsal Capsulodesis and 3-Ligament Tenodesis Technique

**DOI:** 10.1097/PRS.0000000000011489

**Published:** 2024-04-23

**Authors:** Stefanie N. Hakkesteegt, Isabel C. Jongen, Caroline A. Hundepool, Mark J. W. van der Oest, Liron S. Duraku, Reinier Feitz, J. Michiel Zuidam

**Affiliations:** Rotterdam and Amsterdam, the Netherlands; From the 1 Department of Plastic, Reconstructive, and Hand Surgery, Erasmus Medical Center; 2 Hand and Wrist Center, Xpert Clinic; 3 Department of Plastic, Reconstructive, and Hand Surgery, Amsterdam University Medical Center.

## Abstract

**Background:**

Surgical management of midcarpal instability (MCI), also referred to as carpal instability nondissociative, remains controversial because of limited evidence on different techniques. This study aimed to assess and compare differences in patient-reported pain, hand and wrist function, satisfaction, range of motion, and return to work in patients with nontraumatic MCI who underwent surgical treatment either through dorsal wrist capsulodesis or 3-ligament tenodesis (3LT).

**Methods:**

Patients with MCI and persisting complaints after conservative therapy treated with 3LT or dorsal capsulodesis were included. Patients with posttraumatic instability were excluded. Primary endpoints included the Patient Rated Wrist Evaluation and Satisfaction with Treatment Result Questionnaire at 12 months postoperatively. All data were analyzed retrospectively.

**Results:**

A total of 91 patients treated with dorsal capsulodesis and 21 treated with 3LT between December of 2011 and December of 2019 were included. At 12 months postoperatively, both treatment groups reported significant improvements in pain and function scores. However, at 3 months postoperatively, the dorsal capsulodesis group exhibited significantly better outcomes, followed by a greater return-to-work rate (72%) compared with the 3LT group (50%). However, the capsulodesis group demonstrated a decreased range of motion at 3 months that was restored at 12 months postoperatively. No significant difference in satisfaction with treatment was observed.

**Conclusions:**

Both 3LT and dorsal capsulodesis demonstrate promising results for addressing nontraumatic MCI. However, considering the quicker recovery and faster return to work associated with dorsal capsulodesis, the authors recommend favoring capsulodesis over 3LT when both surgical options are deemed suitable for the patient.

The wrist consists of a complex interaction between the carpal bones, radius, and ulna. It derives its stability from numerous intrinsic and extrinsic ligaments, ensuring balance and harmonious movement.^1–3^ The carpal bones form a proximal and distal carpal row, with the proximal row acting as an intercalated segment between the more rigid distal row and the forearm.^1^ In midcarpal instability (MCI), also referred to as carpal instability nondissociative (CIND),^4–6^ laxity of the stabilizing ligaments of the wrist results in malalignment and asynchronous movement of the carpal rows. During radioulnar deviation, the smooth transition of the proximal carpal row from flexion into extension is lost, resulting in a sudden extension known as the “catch-up clunk.”^1,4,5,7^ The abnormal movement of the carpal rows is believed to contribute to the increased focal contact pressures within the wrist, leading to subsequent synovitis, pain, and weakness. MCI is often observed as a manifestation of a generalized ligamentous laxity with complaints unrelated to trauma.^7^ Its diagnosis typically relies on clinical history and physical examination. Provocative tests, such as the Lichtman test, are used to elicit the sensation of the clunk felt by patients.^8^ Imaging studies and arthroscopy may aid in the diagnosis but are generally used to exclude other wrist abnormalities such as intrinsic ligament injuries.^4^ Despite the various proposed grading systems, grading the severity of MCI remains difficult, as its underlying mechanism is still only partly understood.^4,9–12^

Treatment of symptomatic nontraumatic MCI is primarily nonoperative. Prescription of nonsteroidal antiinflammatory medication and static or dynamic splinting may ease complaints, combined with an intensive wrist exercise program led by qualified hand therapists.^13^ Surgical stabilization is recommended if conservative treatment appears insufficient.^6,10,14,15^ Soft-tissue reconstruction techniques used in nontraumatic MCI consist of dorsal wrist capsule enhancement,^9,10,16,17^ augmentation of the existing ligaments,^18,19^ or reconstruction of the affected ligaments.^6,9,20–23^ Dorsal capsulodesis and 3-ligament tenodesis (3LT) are examples of surgical techniques used to treat MCI. The dorsal capsulodesis achieves stability through augmentation of the dorsal capsule, whereas the 3LT procedure focuses on ligament reconstruction using a partial graft of the flexor carpi radialis (FCR).^16,20,21^ Short-term results of the dorsal capsulodesis in the treatment of midcarpal instability seem promising; however, long-term results are lacking.^4^ Although initially described in the management scapholunate dissociation, the 3LT procedure stabilized not only the scapholunate joint but also the midcarpal joint.^21^ This stabilization is thought to result in lasting stability, facilitated by the bone tunnel passing through the scaphoid. However, no definite surgical treatment guidelines have been established, as only short-term data are available and results are based primarily on relatively small case series.^4,9^ Surgical treatment therefore often depends on the surgeon’s preference.

This study aimed to clarify the matter, by evaluating and comparing the dorsal capsulodesis to the 3LT technique in treating nontraumatic MCI. Because few data are available on the postoperative outcomes of these techniques in this specific group of patients, we evaluated patient-reported pain, hand and wrist function, satisfaction, range of motion (ROM), and return to work (RTW) in patients treated with either dorsal capsulodesis or 3LT.

## PATIENTS AND METHODS

In this cohort study, all data have been prospectively collected. Patients treated with dorsal capsulodesis or 3LT between December of 2011 and October of 2019 who completed the 12-month follow-up questionnaire were considered eligible for inclusion. After diagnosis with MCI, hand therapy is initiated for a minimum of 3 months following standardized treatment protocol with regular checkups.^13^ Patients are referred back to the hand surgeon to discuss surgical treatment if nonsurgical treatment provides insufficient improvement. The choice of surgical treatment (dorsal capsulodesis or 3LT) was based on the surgeon’s preference and experience rather than patient characteristics. Electronic patient files were assessed on the presence of MCI with persisting complaints after conservative therapy. Patients were included if they presented with a history of hypermobile joints or when midcarpal instability was diagnosed with a clinical midcarpal shift test according to Lichtman et al.^24,25^ If the continuity of the affected carpal ligaments was questioned, wrist arthroscopy was performed. Patients with a scapholunate interosseus ligament (SLIL) Geissler grade 4 were excluded.^26^ Additional imaging (magnetic resonance imaging or radiography) was not routinely performed but only to exclude other wrist abnormalities. Other exclusion criteria included the following: symptoms of midcarpal instability following a traumatic event, osteoarthritis of the treated wrist, joint or ligament laxity not confirmed by a hand surgeon or hand therapist, chondropathy of the carpal bones, and other anatomical anomalies of the wrist. Patients treated with both dorsal capsulodesis and 3LT were excluded. All surgeons performing dorsal capsulodesis or 3LT were high-volume surgeons, with level 3 to 5 expertise,^27^ and certified according to the Federation of European Societies for Surgery of the Hand.

All patients gave written informed consent before study participation.^28^ Patients were asked before surgery to participate in a routine outcome measurement system implemented at the clinic. The system consists of multiple electronically sent questionnaires, to evaluate risk factors at baseline, and patient-reported outcomes at baseline and 3 and 12 months postoperatively.

### Technique Dorsal Capsulodesis

**Fig. 1.**
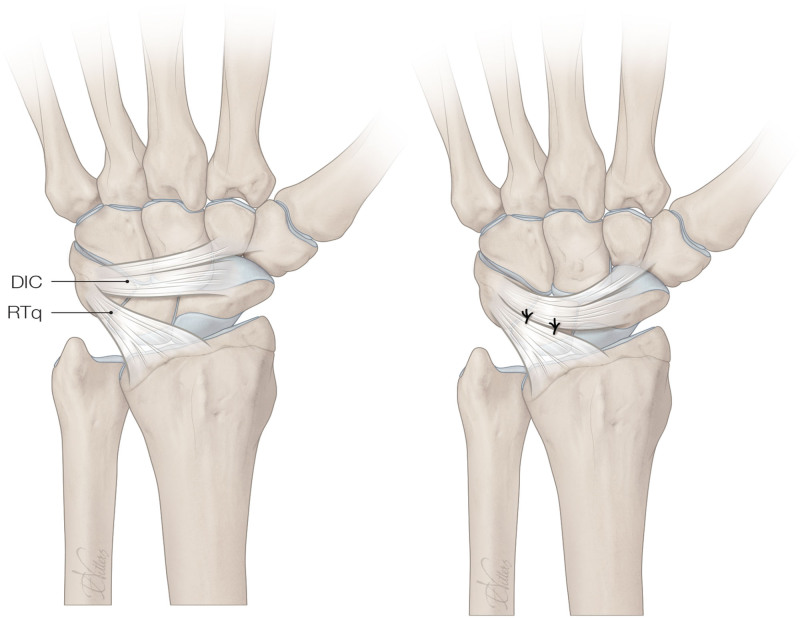
The dorsal capsulodesis technique. *DIC*, dorsal intercarpal ligament; *RTq*, radiotriquetral ligament.

An incision is made on the dorsum of the wrist and the third and fourth compartments were incised longitudinally, both only partially opened in their most distal third part. A neurectomy of the posterior interosseous nerve (PIN) is performed. The wrist capsule is opened and synovectomy is performed. Next, a ligament-sparing capsulotomy is performed by suturing the distal part of the dorsal intercarpal ligament and the radiotriquetral ligament toward each other (or plication), thereby strengthening the dorsal capsule (Fig. 1).

### 3LT Technique

**Fig. 2.**
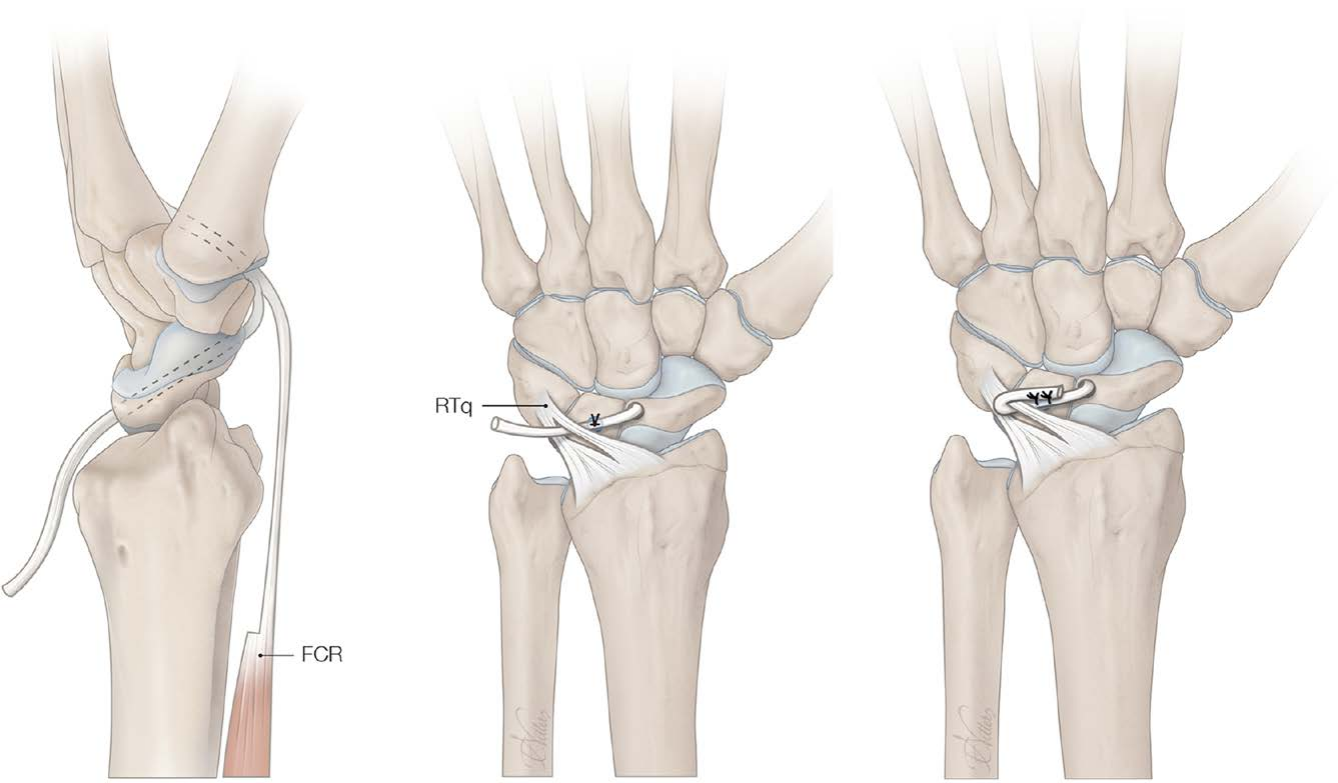
The 3LT technique. *RTq*, radiotriquetral ligament.

The SLIL is dorsally approached through an incision near the fourth extensor compartment base of the wrist. A neurectomy of the PIN is performed. A small volar incision is made over the FCR tendon at the level of the palmar wrist crease to approach the SLIL. A 2.7- or 3.0-mm drill hole (surgeon’s preference) is made through the scaphoid following the longitudinal axis. A strip of the FCR tendon is passed from the volar surface of the scaphoid tuberosity to the dorsal side and fixated with a bone anchor to the lunate (Fig. 2). Next, the FCR tendon strip is passed through the radiotriquetral ligament and sutured back on itself under tension with the wrist in a neutral position.^21^

### Postoperative Rehabilitation

After surgical treatment, patients receive a cast for 3 to 5 days. Next, a splint is made, tendon movements are evaluated and optimized, and load capacity is assessed. Patients are scheduled for a wrist rehabilitation program with treatment and guidance by a certified hand therapist for at least 1 to 3 times a week. Following dorsal capsulodesis, active mobility exercises are started at 2 weeks postoperatively and focus on dorsal and palmar flexion. After 3LT, only passive mobility exercises are started until 4 weeks postoperatively. Starting from weeks 4 to 6, active mobility exercises are initiated in line with the capsulodesis group. At 5 to 6 weeks postoperatively, patients are instructed to wear the splint only during daytime as a protective measure. At weeks 7 to 12 postoperatively, patients are instructed to limit the use of the splint as much as possible and the wrist rehabilitation program is continued with focus on coordination, strength, and stability.

### Outcome Measures

Primary endpoints included the Patient Rated Wrist Evaluation (PRWE) and the Satisfaction with Treatment Result Questionnaire.^29^ The 15-item PRWE questionnaire evaluates pain and function of the affected wrist, with scores ranging from 0 (no pain or dysfunction) to 10 (severe pain or dysfunction).^30^ Patients completed the PRWE questionnaire preoperatively at 3 and 12 months postoperatively. The Satisfaction with Treatment Result Questionnaire was assessed at 12 months postoperatively and was scored using the 5 following categories: poor, moderate, fair, good, and excellent. Patients with poor, moderate, or fair results were classified as unsatisfied, and patients with good or excellent results, as satisfied.

Secondary endpoints included ROM, RTW, and complications. Patients completed the RTW questionnaire at 6 weeks and at 3, 6, and 12 months postoperatively. Subsequent questionnaires are canceled when a patient returns to work completely. With 7 questions, the RTW questionnaire aims to evaluate the effect of surgery on the timing and extent of the patient’s resumption of work activities.

The ROM was objectively assessed and scored at intake and 3 and 12 months postoperatively by a qualified hand therapist working at the clinic. Wrist flexion and extension, radial and ulnar deviation, and forearm rotation were assessed using a goniometer.

Details on the surgical procedure and additional perioperative procedures and wrist arthroscopy reports were documented. Other patient demographics were derived from electronic patient files and consisted of basic patient characteristics, comorbidities, medical history, medication, smoking, occupational status, and hand dominance. Moreover, details on physical examination, diagnostic workup, and previous surgical treatment of the affected hand/wrist were obtained. Postoperative complications were scored according to the International Consortium for Health Outcomes Measurement Complications in Hand and Wrist conditions tool.^31^

### Statistical Analysis

A chi-square test was used to analyze baseline differences between both study groups. Paired *t* tests were used to compare numerical variables within groups on 2 time points. Unpaired *t* tests were used to determine statistical differences between patients in different treatment groups. A 1-way analysis of variance with repeated measures was used to compare continuous parametric data with more than 2 time points. Univariable survival analysis was performed with the Kaplan-Meier method to analyze the time to RTW, and a log rank test was performed to compare survival curves. Patients were censored in this analysis when they reached retirement age or were lost to follow-up. A Cox proportional hazard model was used to adjust for baseline variables in the RTW analysis. Statistical analysis was performed in R statistical programming version 4.0.3. The confidence interval was set at 95%, and values of *P* < 0.05 were considered statistically significant.

## RESULTS

**Fig. 3.**
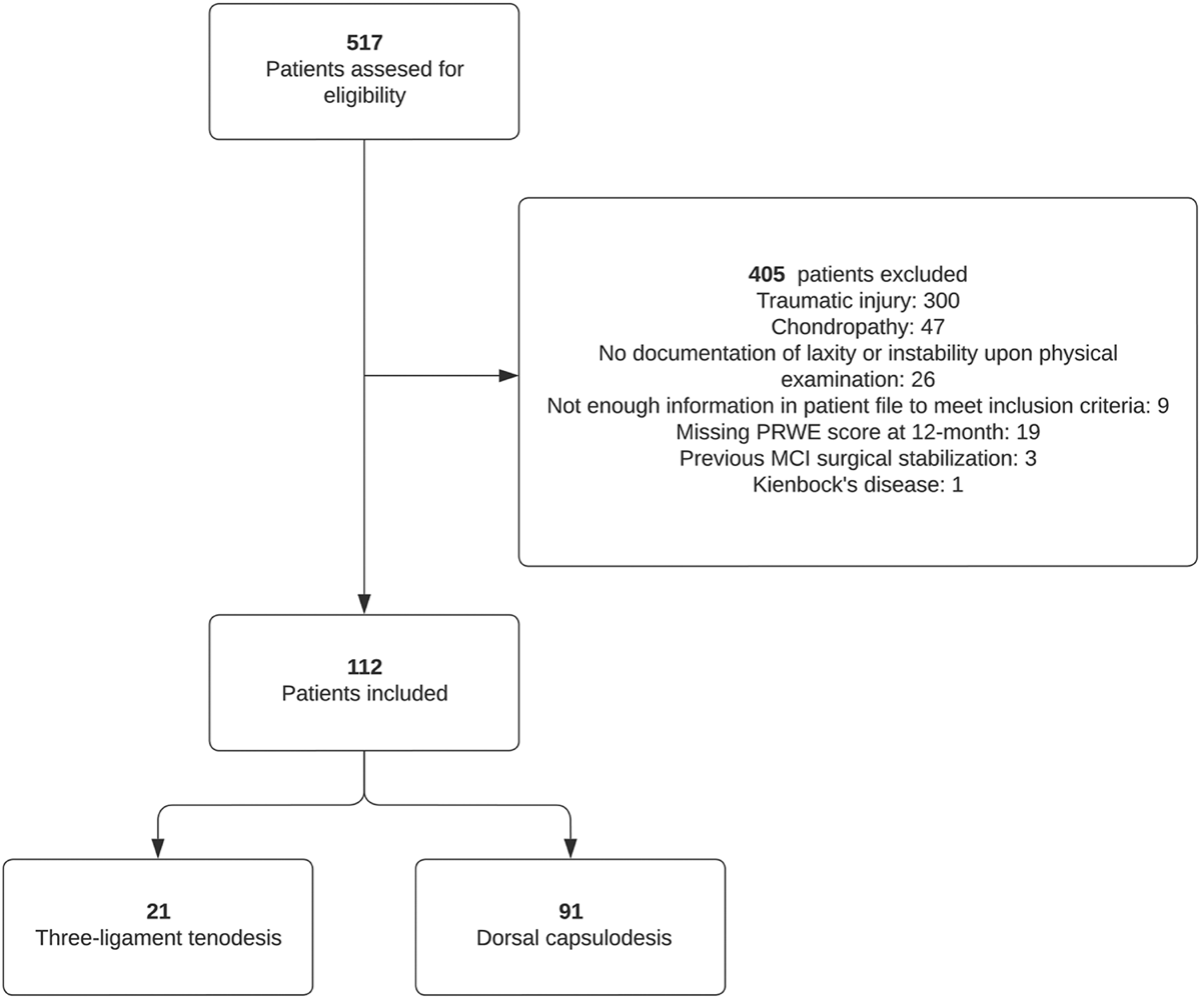
Flow diagram illustrating included and excluded study participants.

A total of 555 patients were treated with 3LT or dorsal capsulodesis in our clinic during the study period. We excluded 443 patients after examination of the patient records because of preceding traumatic injury, no documentation of joint/ligamentous laxity during physical examination, chondropathy of the carpal bones, or previous surgical treatment for MCI. This resulted in the inclusion of 112 patients with nontraumatic wrist laxity treated with either 3LT (*n* = 21) or dorsal capsulodesis (*n* = 91) (Fig. 3 and Table 1). The patient population was predominantly female, with significantly more smokers for the dorsal capsulodesis (*P* = 0.045). The mean age was 32 years for the 3LT group and 30 years for the capsulodesis group (*P* = 0.40) (Table 1). An additional step was incorporated into the procedure in 3 patients treated with 3LT. Following fixation to the lunate, the tendon was routed through the joint capsule distally to the triquetrum and tied to itself. This modification aimed to provide more ulnar support to the midcarpal joint.

**Table 1.** Patient Demographics

	3LT (%)	Dorsal Capsulodesis (%)	*P* ^a^
No. of patients	21	91	
Mean age ± SD, yr	32 ± 10	30 ± 10	0.40
Female sex	16 (76)	82 (90)	0.17
No. of smoking patients	2 (10)	15 (17)	0.049^b^
Mean duration of symptoms ± SD, mo	50.8 ± 54	30.9 ± 43	0.070
Profession			0.014^b^
Light physical work (eg, office work)	5 (24)	24 (26)	
Moderate physical work (eg working in a store)	9 (43)	32 (35)	
Heavy physical work (eg, construction work, road work)	7 (34)	11 (12)	
Hand dominance			0.43
Left	5 (24)	12 (13)	
Right	15 (71)	76 (84)	
Ambidexterity	1 (5)	3 (3)	

a The χ^2^ test was performed for statistical analysis.

b Statistically significant.

### PRWE and Satisfaction with Treatment Result

**Fig. 4.**
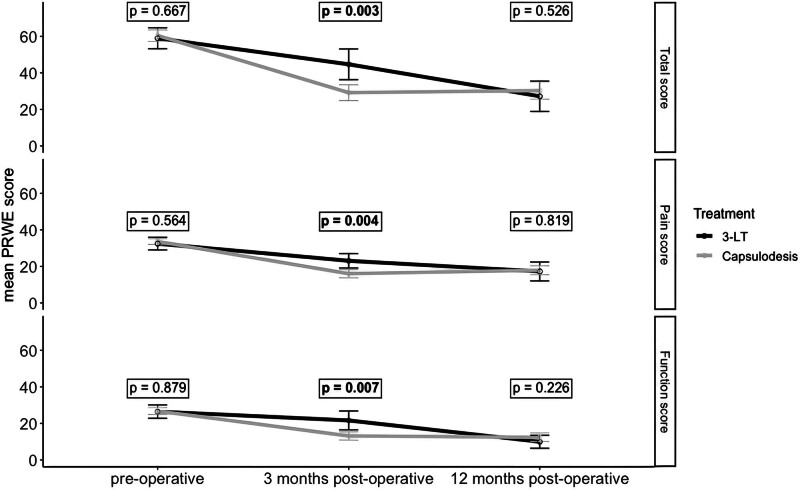
Mean PRWE total, pain, and function scores preoperatively and 3 and 12 months postoperatively with 95% confidence interval error bars for patients treated with 3LT or dorsal capsulodesis. *P* values represent the difference in PRWE scores at intake and 3 and 12 months postoperatively between the 3LT and capsulodesis group. The *gray scales* distinguish between the different surgical treatments.

**Fig. 5.**
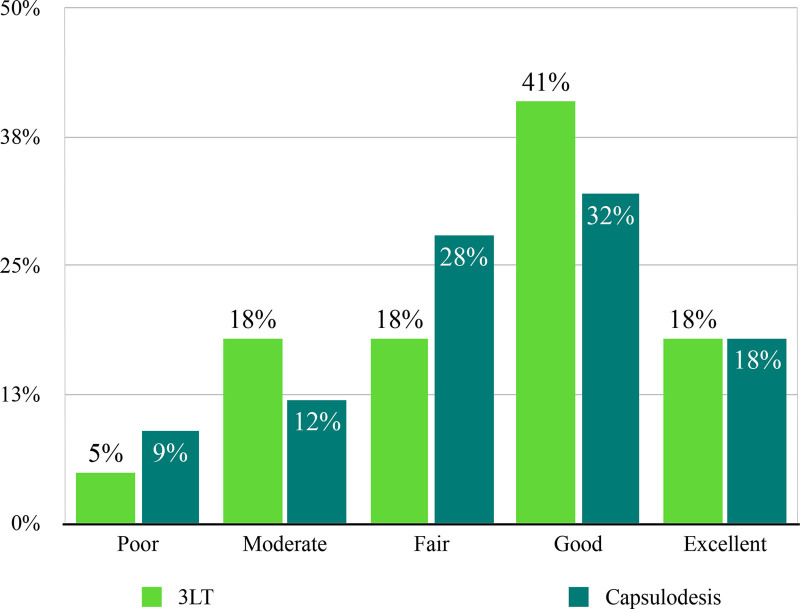
Treatment satisfaction of patients treated with 3LT or dorsal capsulodesis at 12 months postoperatively.

PRWE total scores were similar at intake for the 3LT and capsulodesis groups (58.9 and 60.4 respectively; *P* = 0.67). PRWE function, pain, and total scores improved significantly between intake and 12 months after surgery for both study groups (*P* < 0.05). When comparing both techniques at 3 months postoperatively, PRWE scores were significantly lower for patients treated with dorsal capsulodesis (Fig. 4). However, at 12 months postoperatively, no statistically significant difference was observed (Fig. 4). (**See Table, Supplemental Digital Content 1**, which illustrates the mean PRWE scores and SD at intake and 3 and 12 months after 3LT and dorsal capsulodesis, http://links.lww.com/PRS/H265.) The Satisfaction with Treatment Result Questionnaire at 12 months postoperatively demonstrated a satisfactory treatment result in 59% of patients treated with 3LT and 49% treated with dorsal capsulodesis (*P* = 0.23) (Fig. 5).

### Range of Motion

For dorsal capsulodesis, a significant change in ulnar deviation and palmar and dorsal wrist flexion (*P* < 0.05) was observed, with reduced scores at 3 months postoperatively that restored at 12 months postoperatively. For 3LT, no significant difference was observed (Tables 2 and 3).

**Table 2.** ROM of the Wrist and Forearm in Patients Treated with Dorsal Capsulodesis^a^

Characteristic	Intake (SD)	3 Mo (SD)	12 Mo (SD)	*P*
No.	89	73	25	
Active range of motion, deg				
Dorsal wrist flexion	67 (12)	63 (12)	69 (10)	0.033
Palmar wrist flexion	68 (15)	50 (17)	62 (13)	<0.001
Wrist radial deviation	20 (9)	19 (6)	20 (8)	0.66
Wrist ulnar deviation	32 (9)	31 (8)	33 (8)	0.035
Forearm pronation	80 (9)	80 (8)	79 (8)	0.91
Forearm supination	83 (9)	81 (11)	83 (7)	0.66

a Values presented as mean (SD). A one-way analysis of variance with repeated measures was used. *P* values were calculated for the 25 patients who completed 12-mo follow-up. *P* values represent a difference between 2 of the 3 time points (at intake, 3 mo postoperatively, and 12 mo postoperatively).

**Table 3.** ROM of the Wrist and Forearm in Patients Treated with 3LT^a^

Characteristic	Intake (SD)	3 Mo (SD)	12 Mo (SD)	*P*
No.	21	12	10	
Active range of motion, deg				
Dorsal wrist flexion	61 (16)	47 (11)	60 (9)	0.89
Palmar wrist flexion	63 (18)	39 (12)	52 (15)	0.23
Wrist radial deviation	19 (6)	15 (5)	21 (12)	0.22
Wrist ulnar deviation	34 (10)	26 (9)	31 (10)	0.59
Forearm pronation	79 (8)	76 (12)	79 (10)	0.86
Forearm supination	80 (10)	82 (11)	87 (7)	0.22

a Values presented as mean (SD). A one-way analysis of variance with repeated measures was used. *P* values were calculated for the 10 patients who completed 12-mo follow-up. *P* values represent a difference between 2 of the 3 time points (at intake, 3 mo postoperatively, and 12 mo postoperatively).

### Return to Work

**Fig. 6.**
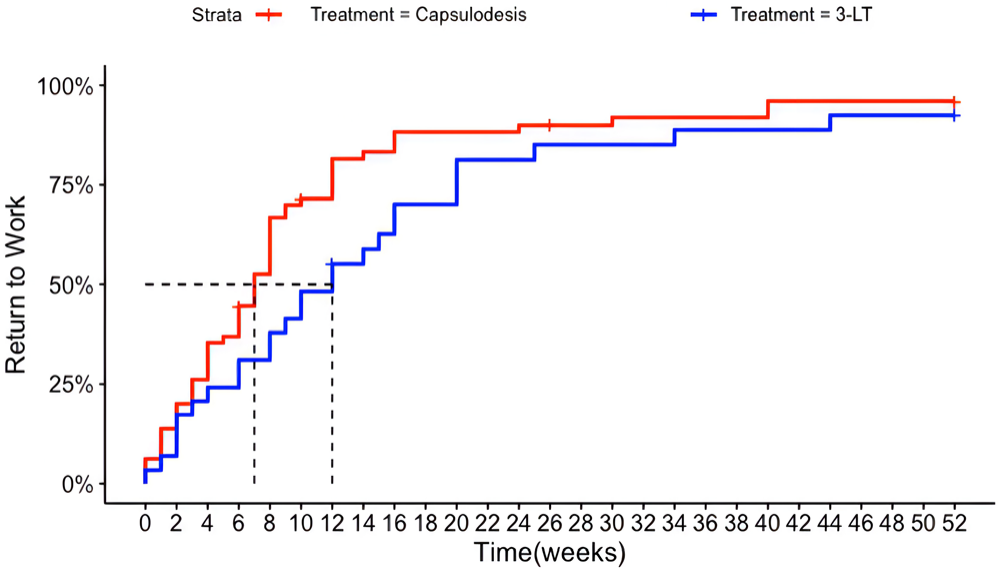
The Kaplan-Meier plot of the RTW in the first 12 months after surgery for patients treated with 3LT or dorsal capsulodesis. The *dotted line* represents the number of weeks postoperatively when 50% of patients have returned to work (mean RTW).

At 3 months postoperatively, the RTW was 72% for patients treated with dorsal capsulodesis and 50% for patients treated with 3LT. The mean RTW was 7 and 12 weeks, respectively (Fig. 6). Most patients had resumed normal working activities 1 year postoperatively (96% and 92%, respectively). There was no significant difference between the 2 treatment curves (*P* = 0.20). As a secondary analysis for RTW, a Cox regression was performed. A hazard ratio of 1.24 (95% CI, 0.80 to 1.94; *P* = 0.33) was found for patients treated with dorsal capsulodesis compared with patients treated with 3LT.

### Complications

Five patients (5%) treated with dorsal capsulodesis had complications that required secondary surgery under local, regional, or general anesthesia (International Consortium for Health Outcomes Measurement Complications in Hand and Wrist grade 3A or 3B). (**See Table, Supplemental Digital Content 2**, which illustrates complications in 5 patients following dorsal capsulodesis requiring revision surgery. *PF*, palmar flexion; *DF*, dorsal flexion, http://links.lww.com/PRS/H266.) After revision surgery, all 5 patients had resolved complaints of instability. No additional surgery was required in the 3LT group. There was no statistically significant difference in complication rates between the groups (*P* = 0.58).

## DISCUSSION

The results of this study show favorable outcomes for both surgical techniques at 12 months postoperatively in the treatment of nontraumatic midcarpal instability. However, PRWE scores were significantly better at 3 months postoperatively for patients treated with dorsal capsulodesis, followed by a greater RTW and a mean RTW 5 weeks shorter than those treated with 3LT. More complications were observed for the dorsal capsulodesis, but this difference was not significant compared with the 3LT.

Comparison of our study outcomes with current literature is difficult because of a scarcity of research in this area, heterogeneity in outcome measures, and inconsistencies in terminology and definitions used for MCI.^1,3,11,21,32^ For instance, the terms midcarpal instability and CIND are used interchangeably in the literature.^7,11^ Midcarpal instability can be further subdivided into palmar MCI (or CIND-VISI), dorsal MCI (or CIND-DISI), combined MCI (or CIND-combined), or extrinsic MCI (or CIND adaptive).^10,11,32^ These differences in terminology lead to unnecessary confusion and contribute to the complexity of deciding on surgical treatment.^11^ Moreover, determining the precise origin of instability remains difficult to discern, as the pathomechanism is still only partly understood and clinical presentation varies greatly between patients.^5,10,14,16,33,34^

To our knowledge, few data are reported on treating nontraumatic MCI with 3LT. One article by Ritt and de Groot^23^ discussed a tenodesis technique using the extensor carpi radialis brevis and reported postoperative improvements in PRWE and visual analogue scale scores in 13 wrists. Krijgh et al.^35^ described a similar hemi–extensor carpi radialis brevis extensor carpi radialis brevis tenodesis technique in patients with Ehlers-Danlos syndrome, reporting decreased pain, improved functionality, and satisfaction with the treatment result. However, improvement scores or preoperative and postoperative scores were not provided. Moreover, we focused on patients with less severe hypermobility complaints and therefore cannot make a comparison to our 3LT results. Von Schroeder^16^ reported on the efficacy of a wrist plication technique for MCI similar to the dorsal capsulodesis technique. This study demonstrated a significant improvement in PRWE total scores consistent with our findings. During an average follow-up duration of 35 months, 2 wrists (7%) required secondary surgery consisting of intercarpal arthrodesis because of recurrent MCI. In our current study, recurrent MCI after dorsal capsulodesis was observed in 3 patients (3%) within 12 months. Recurrences were not observed for 3LT; however, this study group contained fewer patients. Von Schroeder’s cohort included patients with minor and major wrist trauma, making it challenging to identify the origin of instability, as it could be attributed to either trauma or an underlying ligamentous laxity.^34^ In contrast, we ensured that our study groups comprised only patients with MCI resulting from underlying ligamentous laxity; therefore, we believe that our results are applicable to this specific patient population. Interestingly, the complication rates were higher in the capsulodesis group (5%) when compared with the 3LT group (0%), although the difference did not reach statistical significance. This disparity could potentially be attributed to the relatively limited number of patients in the 3LT group, as previous literature described 4 complications in a group of 203 patients treated with 3LT.^36^

Studies focusing on PIN neurectomy show that denervation alone may lead to a decrease in pain and a significant improvement in function.^37–39^ This raises the question of whether the observed decrease in pain and improvement in function after dorsal capsulodesis or 3LT results from joint stability restoration or articular denervation. Further understanding of its potential role is needed before we can decide on a superior surgical treatment. With our data, we could not assess the role of the PIN in the reduction of preoperative symptoms.

Our study has a few limitations. First, hypermobility scores (eg, Beighton scores^40^) and the degree (minor versus gross instability) or location of instability (ie, palmar, dorsal, combined, extrinsic) were not documented for most patients. We therefore could not assess whether outcomes differed between patients with varying grades of hypermobility. Second, patients were asked whether there had been a traumatic injury to the wrist preceding the complaints, which could therefore be potentially subject to recall bias. Third, significantly more patients with a profession requiring heavy physical work underwent the 3LT procedure, possibly impacting the RTW findings. Moreover, differences in postoperative hand therapy regimens might have influenced the RTW outcomes. Lastly, in 14% of 3LT patients, an additional step was incorporated into the procedure. Given the limited number of patients, no subgroup analysis was performed. This is potentially confounding and complicates the interpretation of the 3LT results.

## CONCLUSIONS

Given the study results, we conclude that both dorsal capsulodesis and 3LT are suitable surgical techniques for treating nontraumatic midcarpal instability that is nonresponsive to conservative treatment. Patients in both study groups reported significant improvements regarding pain and function. However, those treated with dorsal capsulodesis demonstrated a quicker recovery compared with 3LT. Consequently, when surgeons face an equally justifiable choice between dorsal capsulodesis and 3LT, the former appears to be a preferable option. Nevertheless, a better understanding of the pathomechanism behind midcarpal instability and the implementation of uniform terminology is essential for achieving optimal surgical results.

## APPENDIX

The members of the Hand-Wrist Study Group are as follows: R. A. M. Blomme, B. J. R. Sluijter, D. J. J. C. van der Avoort, A. Kroeze, J. M. Smit, J. Debeij, E. T. Walbeehm, G. M. van Couwelaar, G. M. Vermeulen, J. P. de Schipper, J. F. M. Temming, J. H. van Uchelen, H. L. de Boer, K. P. de Haas, K. Harmsen, O. T. Zöphel, R. Koch, T. M. Moojen, X. Smit, G. J. Halbesma, R. van Huis, P. Y. Pennehouat, K. Schoneveld, Y. E. van Kooij, R. M. Wouters, P. Zagt, J. Veltkamp, A. Fink, W. A. de Ridder, J. Tsehaie, R. Poelstra, M. C. Janssen, P. O. Sun, V. J. M. M. Schrier, L. Hoogendam, J. S. Teunissen, J Dekker, M. L. Jansen-Landheer, M. H. P. Ter Stege, J. S. Souer, R. W. Selles, H. P. Slijper, S. E. R. Hovius, and R. Feitz.

## DISCLOSURE

The authors have no financial interest to declare in relation to the content of this article. No funding was received for this article.

## Supplementary Material


