# Supplementary material for: Surgical Techniques in Nontraumatic Midcarpal Instability: Evaluating the Dorsal Capsulodesis and 3-Ligament Tenodesis Technique
Source: Plast Reconstr Surg. 2024 Apr 23;155(1):109–18. doi: 10.1097/PRS.0000000000011489 (PMC11651348; doi:10.1097/PRS.0000000000011489)
Supplement: Supplementary file 1 [file prs-155-109e-s001.pdf]

Online Supplementary Material 1. Table that illustrates the mean Patient Reported Wrist Evaluation (PRWE) scores and standard deviation at intake, 3- and 12-months after 3LT and dorsal capsulodesis.

	Intake		3 Months		12 Months	
	3-LT	Dorsal capsulodesis	3-LT	Dorsal capsulodesis	3-LT	Dorsal capsulodesis
PRWE total	58.9 (13.7)	60.4 (15.7)	44.8 (19.5)	29.2 (20.3)	27.2 (19.9)	30.3 (23.2)
PRWE pain	32.5 (8.3)	33.6 (7.8)	23.3 (8.9)	16.0 (10.6)	17.2 (12.4)	17.9 (12.2)
PRWE function	26.5 (8.7)	26.8 (9.3)	21.6 (10.6)	13.1 (8.9)	10.0 (8.5)	12.4 (11.5)
