# Supplementary material for: Surgical Techniques in Nontraumatic Midcarpal Instability: Evaluating the Dorsal Capsulodesis and 3-Ligament Tenodesis Technique
Source: Plast Reconstr Surg. 2024 Apr 23;155(1):109–18. doi: 10.1097/PRS.0000000000011489 (PMC11651348; doi:10.1097/PRS.0000000000011489)
Supplement: Supplementary file 2 [file prs-155-109e-s002.pdf]

Online Supplementary Material 2. Table that illustrates complications in five patients following dorsal capsulodesis requiring revision surgery.

	Age	Sex	Reason for revision	Operative procedure	Postoperative outcome
1.	23	F	Pain, stiffness, DF/PF -50/25, scar tissue adhesions	Tenolysis extensor tendons	Increased ROM, overall good postoperative result
2.	27	F	Persistent complaints of instability, pain during flexion, PF 40, synovitis extensor tendons	Redo dorsal capsulodesis and synovectomy extensor tendons	Good postoperative result
3.	35	F	Reduced ROM, specifically PF	Release capsulodesis	Good postoperative result, improved ROM
4.	38	F	Postoperative pain due to synovitis extensor tendons	Synovectomy extensor tendons	Good postoperative result
5.	33	F	Postoperative pain and recurrent dorsal wrist ganglion	Excision ganglion, synovectomy extensor tendons, and redo dorsal capsulodesis	Good postoperative result

PF, palmar flexion; DF, dorsal flexion.
